# Prognostischer und differenzialdiagnostischer Stellenwert der Liquordiagnostik bei neurodegenerativen Demenzerkrankungen

**DOI:** 10.1007/s00115-022-01339-6

**Published:** 2022-06-07

**Authors:** R. Haußmann, P. Homeyer, M. D. Brandt, M. Donix

**Affiliations:** 1 Klinik und Poliklinik für Psychiatrie und Psychotherapie, Universitätsklinikum Carl Gustav Carus, Technische Universität Dresden, Fetscherstr. 74, 01307 Dresden, Deutschland; 2 Klinik und Poliklinik für Neurologie, Universitätsklinikum Carl Gustav Carus, Technische Universität Dresden, Fetscherstr. 74, 01307 Dresden, Deutschland; 3 DZNE, Deutsches Zentrum für Neurodegenerative Erkrankungen, Dresden, Deutschland

**Keywords:** Liquoranalytik, Alzheimer-Demenz, Lewy-Körperchen-Erkrankung, Frontotemporale Demenz, Neurodegenerative Erkrankungen, Cerebrospinal fluid analysis, Alzheimer’s disease, Lewy body dementia, Frontotemporal dementia, Neurodegenerative disease

## Abstract

Die Liquordiagnostik im Rahmen von Demenzerkrankungen ist trotz neuer diagnostischer Möglichkeiten im Bereich der PET(Positronen-Emissions-Tomographie)-Bildgebung weiterhin von hoher klinischer Relevanz. Insbesondere für die Alzheimer-Erkrankung existieren validierte Biomarker, die die Diagnose untermauern und bei der diagnostischen Abgrenzung anderer Demenzätiologien hilfreich sein können. Während unauffällige Liquorbefunde mit negativen Demenz- und Destruktionsmarkern die überwiegende Mehrzahl neurodegenerativer Demenzursachen mit hoher diagnostischer Sicherheit ausschließen, stellen in der klinischen Praxis vor allem überlappende Biomarkerprofile bei primär neurodegenerativen Demenzursachen ein substanzielles Problem bei der Befundinterpretation dar. Deshalb bedarf die Liquorbefundinterpretation stets einer kontextualisierten Betrachtung unter Würdigung der klinischen Symptomatik und Verlaufscharakteristika des entsprechenden demenziellen Syndroms. Außerdem stellen auch Mischbefunde eine häufige diagnostische Herausforderung dar, für deren Interpretation es profunder Kenntnisse im Bereich von Präanalytik, möglicher Liquorbefundkonstellationen und natürlich der verschiedenen in Betracht kommenden Demenzätiologien bedarf. Auch Liquorbiomarker für Synukleinopathien, Tauopathien sowie TDP43(Transactive response DNA binding protein 43 kDa)-Proteinopathien sind Gegenstand aktueller Untersuchungen, wenngleich diese noch nicht den Weg in die klinische Routinediagnostik gefunden haben.

## Abstract (en)

Cerebrospinal fluid (CSF) analysis is an important diagnostic tool in the assessment of dementia. For the differentiation of Alzheimer’s disease from other etiologies of dementia syndromes, established biological markers could be helpful to confirm a distinctive neuropathology. Whereas negative CSF findings can rule out the majority of primarily neurodegenerative disorders, overlapping biomarker profiles remain a diagnostic challenge. Therefore, it is important to interpret CSF results within a specific clinical context. Furthermore, atypical CSF data can be challenging and require profound knowledge of preanalytics, biomarker profiles and the broad spectrum of diseases associated with cognitive decline. Beyond the Alzheimer’s disease clinical spectrum, current studies aim at investigating CSF biomarkers to better differentiate tauopathies, TDP43(Transactive response DNA binding protein 43 kDa)-proteinopathies and synucleinopathies.

## Einleitung

Im Bereich der Demenzdiagnostik und insbesondere bei der ätiologischen Zuordnung demenzieller Syndrome kommen der Liquordiagnostik zwei wesentliche Funktionen zu. Unter negativdiagnostischem Aspekt dient sie vorrangig dem Ausschluss entzündlicher Ursachen (z. B. Virusenzephalitiden, Lues, Morbus Whipple, Neuroborreliose, Neurosarkoidose, Vaskulitiden, Paraneoplasien) und, im Falle rasch progredienter demenzieller Syndrome, dem Ausschluss von Prionenerkrankungen, während sie im Sinne einer Positivdiagnostik die Diagnosestellung einer (frühen) Alzheimer-Erkrankung und anderer neurodegenerativer Demenzätiologien unterstützen kann [7]. Ferner wird die Liquordiagnostik in der klinischen Praxis auch genutzt, um eine neurodegenerative Genese neurokognitiver Defizite mit demenzieller Beeinträchtigungsschwere auszuschließen [35]. Vaskuläre Läsionen, alkoholassoziierte kognitive Störungen, der idiopathische Normaldruckhydrozephalus, chronische Subduralhämatome und auch funktionelle kognitive Störungen stellen beispielsweise mögliche nicht neurodegenerative Demenzursachen dar.

Gemäß konsentierten Leitlinienempfehlungen sollten Zellzahl, Gesamtprotein, Laktat und Glukose, der Albuminquotient sowie die intrathekale IgG-Produktion inkl. oligoklonaler Banden bestimmt werden [7]. Je nach klinischer Indikation kann unter Umständen auch die Bestimmung der intrathekalen IgA- und IgM-Produktion sinnvoll sein.

Die demenzspezifische neurochemische Liquordiagnostik hat einen unbestrittenen Wert in der Erstdiagnostik klinisch unklarer Fälle und insbesondere in der Differenzierung neurodegenerativer und nicht neurodegenerativer Demenzursachen [7]. Dabei wird stets die kombinierte Bestimmung der Parameter beta-Amyloid-1-42 (Aβ42), beta-Amyloid-1-40 (Aβ40), Gesamt-Tau und Phospho-Tau (pTau) empfohlen, da diese der Bestimmung einzelner Parameter überlegen ist. Metaanalytische Daten zeigen beispielsweise, dass die isolierte Aβ42-Bestimmung unzureichend in der Differenzierung verschiedener Demenzentitäten ist und das Risiko falsch-positiver Alzheimer-Demenz-Diagnosen erhöht [21]. Darüber hinaus weisen weder die isolierten Gesamt-Tau- noch die isolierten pTau-Werte ausreichende Sensitivitäten und Spezifitäten auf, um beim Patienten mit leichter kognitiver Störung („mild cognitive impairment“ [MCI]) eine Konversion zur Alzheimer-Demenz zu prognostizieren [32]. Die Daten zeigen, dass ein negativer Liquorbefund zwar mit großer diagnostischer Sicherheit eine Alzheimer-Erkrankung ausschließt, ein positiver Liquorbefund das Vorliegen einer Alzheimer-Demenz jedoch nicht zwingend bestätigt. Zurückzuführen ist dies auf die altersabhängig hohe Prävalenz einer Amyloidpathologie, sodass eine neurodegenerative Komorbidität (z. B. in Form einer Lewy-Körperchen-Demenz) auch bei Alzheimer-typischer Liquorkonstellation möglich ist [32]. Vor diesem Hintergrund erfolgt die ätiologische Zuordnung des Demenzsyndroms in erster Linie nach klinischen Kriterien und kann liquordiagnostisch gestützt werden. Im Prodromalstadium einer Alzheimer-Demenz hingegen ist die biomarkerbasierte ätiologische Zuordnung kognitiver Defizite aufgrund der pathophysiologischen Kaskade (Amyloid- und Tau-Pathologie geht der substanziellen Neurodegeneration und alltagsrelevanten kognitiven Beeinträchtigung um Jahre voraus) den klinischen und bildgebenden Kriterien überlegen.

In der Differenzierung verschiedener neurodegenerativer Demenzursachen ist der diagnostische Wert dieser Parameter jedoch sehr begrenzt und gemäß der aktuellen S3-Leitlinie beispielsweise auch nicht ausreichend, um eine vaskuläre Demenz von neurodegenerativen Demenzursachen abzugrenzen [7].

Der Fokus dieser Literaturübersicht ist die Darstellung der aktuellen Evidenz zum diagnostischen Wert etablierter und experimenteller Neurodegenerations- und Demenzparameter im Liquor bei verschiedenen neurodegenerativen Demenzätiologien.

## Methodik

Diese Übersichtsarbeit wurde unter Nutzung der über PubMed verbundenen biomedizinischen Datenbanken erstellt. Eine zusätzliche Suche erfolgte anhand der in den Literaturverzeichnissen gefundenen Original- und Übersichtsarbeiten. Dabei wurden präferenziell Originalarbeiten gesucht. Auch die verfügbaren Leitlinien wurden berücksichtigt. In Anbetracht der heterogenen Datenlage fanden verschiedene Studiendesigns und auch Literaturübersichten Berücksichtigung.

## Liquorparameter bei der Alzheimer-Erkrankung

In der aktuellen Literatur werden das Aβ42-Peptid, das Tau- sowie das pTau-Protein als sogenannte „core biomarkers“ bezeichnet, die im Rahmen der Alzheimer-Erkrankung charakteristische Veränderungen aufweisen [29]. Während das Aβ42-Peptid aufgrund extrazellulärer Ablagerungen in Form von Amyloidplaques eine verminderte Konzentration im Liquor aufweist, sind die Konzentrationen der intrazellulären Proteine Tau und pTau typischerweise erhöht. In diesem Zusammenhang ist die Tau-Protein-Konzentration mit der unspezifischen Degeneration kortikaler Neuronen und die pTau-Protein-Erhöhung mit der spezifischeren Bildung intrazellulärer neurofibrillärer „tangles“ assoziiert [29]. Die pTau-Protein-Konzentrationserhöhung ist daher relativ spezifisch für das Vorliegen einer Alzheimer-Erkrankung [39, 40, 44] und differenziert gut zwischen Gesunden und Erkrankten (Sensitivität 74 %, Spezifität 92 %; [11]). Die isolierte Tau-Erhöhung ist Ausdruck eines degenerativen Prozesses und bildet somit eher morphologische Veränderungen als eine krankheitsspezifische Neuropathologie ab. Auch eine Aβ42-Erniedrigung im Liquor differenziert Gesunde und Erkrankte mit relativ hoher Sicherheit (Sensitivität 86 %, Spezifität 89 %; [11]). Insbesondere das gleichzeitige Vorliegen veränderter Amyloid- und Tau-Marker im Liquor ist mit hoher Sensitivität (89 %) und Spezifität (90 %) bei der Differenzierung von Gesunden und Erkrankten assoziiert [2]. Durch die Bestimmung der „pTau/Aβ42 ratio“ kann die Spezifität dieser Differenzierung weiter erhöht werden (Sensitivität 86 %, Spezifität 90 %; [24]). Als weiteres Verhältnis hat sich die Bestimmung der „Aβ42/40 ratio“ etabliert, welche wesentliche Aspekte des Entstehungsmechanismus der mutmaßlich krankheitsauslösenden Amyloidplaques berücksichtigt. Die Konzentration von freien Aβ42-Peptiden hängt nicht nur von deren Verbrauch im Rahmen der Plaquebildung ab, sondern auch von der Menge des gebildeten Amyloid-Precursor-Proteins (APP), welches u. a. in die Peptide Aβ40 und Aβ42 gespalten wird. Somit spiegelt ein in Relation zur Aβ40-Konzentration erniedrigter Aβ42-Gehalt im Liquor noch genauer die pathologische Plaquebildung wider. Passend dazu weist die „Aβ42/40 ratio“ bessere Korrelationen mit Plaques in Amyloid-PET(Positronen-Emissions-Tomographie)-Befunden auf, weshalb eine pathologisch erniedrigte „Aβ42/40 ratio“ in der Befundinterpretation letztlich bedeutsamer ist als eine isolierte Aβ42-Erniedrigung [19, 46].

Der prototypische Liquorbefund im Rahmen einer Alzheimer-Erkrankung sind eine erniedrigte Aβ42-Peptid-Konzentration bzw. eine erniedrigte „Aβ42/40 ratio“ sowie erhöhte Tau- und pTau-Konzentrationen. In der klinischen Praxis liegen häufig Mischbefunde vor, die selbst für erfahrene Kliniker eine Herausforderung in der kontextualisierten Befundinterpretation sind [46]. Ein wesentlicher Grund für die Häufigkeit von Mischbefunden ist die Tatsache, dass die Symptome einer Alzheimer-Erkrankung nicht vollständig spezifisch sind und somit stets auch andere neurodegenerative oder vaskuläre Pathologien diagnostisch in Betracht kommen [33]. In diesem Zusammenhang zeigen Amyloid-PET-Daten beispielsweise, dass etwa 12 % der Patienten mit klinischem Alzheimer-Syndrom keine zerebrale Amyloidpathologie aufweisen. Aufgrund der ätiologischen Heterogenität des MCI-Syndroms ist die klinische Spezifität im Prodromalstadium noch geringer, sodass sich bei 50–60 % aller Patienten mit MCI keine biomarkerbasierten neuropathologischen Korrelate finden lassen [33]. Hieraus lässt sich aber im Umkehrschluss eine besondere Bedeutung der Biomarkerdiagnostik in der Frühphase ableiten, da Alzheimer-spezifische neuropathologische Veränderungen dem vollständigen charakteristischen klinischen Syndrom um Jahre vorausgehen.

Gemäß leitlinienbasierten Empfehlungen bedarf es stets einer kombinierten Bestimmung der „Aβ42/40 ratio“ sowie der Tau-Proteine [7, 46]. Die isolierte Bestimmung des Aβ42-Peptids unterliegt einer hohen biologischen Varianz und ist teilweise anfällig für präanalytische Fehler, vorrangig durch selektive Anhaftungen an die Oberfläche bestimmter Probenröhrchen [20, 46]. Während die Verwendung standardisierter Probenröhrchen hier die Präanalytik verbessern hilft, ist die mangelnde Standardisierung der Analytik selbst für die Vergleichbarkeit der Messwerte von verschiedenen Laboren weiterhin ein Problem. Obwohl eine Tau-Protein-Erhöhung als unspezifischer Neurodestruktionsmarker gewertet wird, kommt sie bei neurodegenerativen Erkrankungen interessanterweise nahezu ausschließlich bei der Alzheimer-Erkrankung vor, sodass das Gesamt-Tau eine hohe Spezifität, aber eine geringe Sensitivität aufweist [16]. Hohe Tau- und pTau-Konzentrationen sind im Vergleich zur „Aβ ratio“ außerdem mutmaßlich bessere Marker der Krankheitsaktivität und weisen deutliche Assoziationen mit einer schnelleren Krankheitsprogression auf [47]. Auch neuere Arbeiten bestätigen, dass hohe Tau-Konzentrationen mit einem rasch progredienten Verlauf einer Alzheimer-Erkrankung assoziiert sind [6]. Auf die Möglichkeit einer abfallenden Tau-Protein-Konzentration im Erkrankungsverlauf vaskulärer Demenzen sei verwiesen, da dies bei keiner anderen neurodegenerativen Demenzerkrankung auftritt und für das Verlaufsmonitoring interessant sein kann. Allerdings trifft dies nur für akute vaskuläre Ereignisse zu, wo in der Regel bereits die zeitliche Assoziation zum Beginn der kognitiven Defizite wegweisender Hinweis auf die Ätiologie ist. Bei der häufigeren und klinisch herausfordernden Differenzierung zwischen chronisch vaskulären Veränderungen in Form einer zerebralen Mikroangiopathie und primär neurodegenerativer Genese ist kein ausreichender Mehrwert auf Grundlage der Tau-Bestimmung im Liquor zu erwarten.

Robuste Assoziationen zwischen synaptischer Dichte und kognitiven Defiziten bei der Alzheimer-Erkrankung begründen, weshalb auch Biomarker der synaptischen Neurodegeneration erstrebenswert sind. Bei Neurogranin handelt es sich um ein postsynaptisches Protein, das insbesondere in gedächtnisbildenden Hirnstrukturen exprimiert wird und als vielversprechender synaptischer Neurodegenerationsmarker zählt [26]. Insbesondere bei der Alzheimer-Erkrankung vom hippocampalen Typ, aber auch bei MCI mit hohem Konversionsrisiko werden erhöhte Neurograninkonzentrationen im Liquor gemessen [26].

## Liquorbiomarkerbestimmung bei Patienten mit MCI

Die oben genannten „core biomarkers“, die zur Differenzierung gesunder Personen und Patienten mit Alzheimer-Erkrankung geeignet sind, differenzieren auch Patienten mit MCI, bei denen längsschnittliche Progredienz ätiologisch auf eine Alzheimer-Erkrankung zurückzuführen ist (AD-MCI), von Patienten mit einer nicht neurodegenerativen MCI-Genese [29]. Hinsichtlich der Liquorbiomarkerbestimmung bei Patienten mit MCI zur ätiologischen Zuordnung, zur Abschätzung des Konversionsrisikos und damit zur Demenzprädiktion existieren jedoch bislang keine leitlinienbasierten Empfehlungen. Bevor die Liquorbiomarkerbestimmung in derartigen Konstellationen erwogen wird, bedarf es neben einem ausführlichen Beratungsgespräch zunächst der testpsychometrischen Objektivierung des MCI-Syndroms, dessen testpsychometrischer Abgrenzung zu altersphysiologischen Gedächtnisdefiziten und des Ausschlusses möglicherweise behandelbarer Ursachen mittels Schädel-MRT, einer umfassenden laborchemischen Untersuchung, eines Ausschlusses relevanter psychiatrischer Komorbiditäten und der Überprüfung der Medikation hinsichtlich potenziell dyskognitiv wirkender Medikamente [33, 37]. Auch und insbesondere beim Patienten mit MCI sind die kontextualisierte Befundinterpretation und eine konsequente Fahndung nach konkurrierenden Ätiologien des MCI-Syndroms bedeutsam. Eine alleinig biomarkerbasierte Risikovorhersage ist, aufgrund der unzureichenden Spezifität und der nichtlinearen Zusammenhänge zwischen Alter, kognitiver Verschlechterung und Amyloidpathologie, nicht statthaft [33]. Diesbezüglich sind insbesondere Sättigungseffekte im höheren Lebensalter zu berücksichtigen, die mit einer altersabhängig weiter abnehmenden Spezifität derartiger Liquorparameterveränderungen verbunden sind. Ferner sind die formal (aktuell noch) fehlende therapeutische Konsequenz im Falle einer AD-MCI gemäß Zulassungsstatus von Antidementiva sowie psychische Begleitreaktionen und sozial- oder versicherungsrechtliche Konsequenzen bei dieser Entscheidung zu würdigen [33].

Zur Einschätzung des Risikos einer zugrunde liegenden Alzheimer-Erkrankung beim Patienten mit MCI-Syndrom wurde die binäre ATN-Klassifikation geschaffen, welche das Alzheimer-Konversionsrisiko anhand von Amyloid- („Aβ ratio“ oder Amyloid-PET), Tau- (Tau-PET oder pTau-Protein im Liquor) und Neurodegenerationsmarkern (Tau-Protein im Liquor, Temporallappen- oder Hippocampusatrophie, Hypometabolismus im FDG-PET) beschreibt [17, 33]. Je nach vorliegender Biomarkerkonstellation variiert das längsschnittliche Risiko für die Entwicklung einer Alzheimer-Erkrankung (Nachweis pathologischer Amyloid- und Tau-Marker: hohe Wahrscheinlichkeit von 90 %/5 Jahre; Nachweis pathologischer Amyloid- oder Tau-Marker: mittlere Wahrscheinlichkeit von 45 bis 50 %/5 Jahre; weder auffällige Amyloid- noch Tau-Marker: geringe Wahrscheinlichkeit von 10 %/5 Jahre; [33]). Von besonderer Aussagekraft hinsichtlich der Prädiktion einer Konversion von der MCI zur Alzheimer-Demenz scheint auch eine erhöhte „pTau/Aβ42 ratio“ zu sein [4]. Von zweifelsfreiem diagnostischem Wert sind Tau und pTau in der Abgrenzung von neurodegenerativ und rein affektiv bedingten MCI [36].

Darüber hinaus sind erhöhte Neurofilamentkonzentrationen im Liquor mit einer schnellen Abnahme des MMST-Werts und rascheren strukturellen Hirnveränderungen bei MCI-Patienten vergesellschaftet [48]. Dies bedeutet, dass erhöhte Neurofilamentkonzentrationen im Liquor gerade zu Beginn des Erkrankungsverlaufs einen wichtigen Parameter darstellen, um zwischen einer neurodegenerativen und einer nichtneurodegenerativen Demenzerkrankung zu unterscheiden.

## SNAP-Konzept

Bei „suspected non-Alzheimer’s pathophysiology“ (SNAP) handelt es sich um ein biomarkerbasiertes Konzept zur Charakterisierung von kognitiv Gesunden und Patienten mit MCI mit unauffälligen Amyloid- und positiven Neurodegenerationsmarkern. Als mögliches SNAP-Korrelat werden andere Pathomechanismen wie TDP43(Transactive response DNA binding protein 43 kDa)-Pathologie und/oder Hippocampussklerose, welche klinisch kaum von einer Alzheimer-Erkrankung zu differenzieren sind, angenommen [17]. Auch physiologische Alterungsprozesse im medialen Temporallappen werden diskutiert („primary age-related tauopathy“ [PART]; [18]). Das klinische Progressionsrisiko dieser Patienten wird als intermediär und geringer als bei präklinischer Alzheimer-Erkrankung angenommen [18]. Die Kenntnis dieser möglichen Liquorkonstellation kann insbesondere in der Beratung von Patienten hinsichtlich prognostischer Implikationen hilfreich sein.

## Differenzialdiagnostischer Wert der Liquordiagnostik hinsichtlich anderer neurodegenerativer Demenzätiologien

### Parkinson- und Lewy-Körperchen-Demenz

Insbesondere bei Synukleinopathien wie der Parkinson- und der Lewy-Körperchen-Demenz ist die klinische Diagnosestellung aufgrund großer klinischer und pathologischer Überlappungen mit anderen neurodegenerativen Demenzerkrankungen anspruchsvoll, weshalb der Liquordiagnostik auch bei diesen Demenzätiologien eine hohe klinische Bedeutung zukommt [31]. Im Vergleich zu den etablierten Alzheimer-Biomarkern im Liquor zeigt die Aβ42-Peptid-Erniedrigung auch hier robuste Zusammenhänge mit der Entwicklung bzw. einem Progress kognitiver Defizite im Rahmen der Parkinson- und Lewy-Körperchen-Demenz [31]. In der differenzialdiagnostischen Abgrenzung zwischen einer Alzheimer-Erkrankung und der Lewy-Körperchen-Demenz kommen dem Tau- und dem pTau-Protein sowie der „Aβ42/Aβ38 ratio“ eine potenzielle Bedeutung zu [31]. Bei der Befundinterpretation gilt es jedoch, die großen Überlappungen der Liquorbiomarker bei Alzheimer-Erkrankung und Lewy-Körperchen-Demenz zu bedenken [25]. In einer multizentrischen Kohortenstudie mit 417 Patienten mit klinischer Diagnose einer Lewy-Körperchen-Demenz konnte beispielsweise gezeigt werden, dass Alzheimer-typische Liquorbefunde bei dieser Demenzätiologie häufig sind und die Positivität für Amyloid- und Tau-Biomarker im Liquor insbesondere mit steigendem Alter zunimmt [9]. Hinsichtlich der Liquorbefunde wiesen 39 % der Patienten weder Amyloid noch Tau-Biomarker auf, 32 % zeigten isoliert auffällige Amyloid- und 15 % sowohl pathologische Amyloid- als auch auffällige Tau-Biomarker und 13 % isolierte Auffälligkeiten der Tau-Biomarker. Insbesondere ApoE4-Allelträger wiesen mit steigendem Alter häufiger auffällige Amyloidmarker auf, welche, synergistisch mit erhöhten Tau-Biomarkern, der wesentliche Prädiktor für einen kognitiven Abbau waren. Ferner korrelierten auffällige Tau-Biomarker mit einer geringeren Prävalenz von Parkinson-Syndromen und REM-Schlaf-Verhaltensstörungen [9]. Diese Befunde deuten darauf hin, dass durchaus eine Komorbidität verschiedener neurodegenerativer Erkrankungen vorliegen kann und entsprechend das klinische Bild prägt. Inwieweit kombinierte neuropathologische Veränderungen auf Biomarkerebene prädiktive Marker sind, ist in Ermangelung der Verfügbarkeit valider Verfahren zum labordiagnostischen Nachweis einer Synukleinopathie noch nicht hinreichend geklärt. Diesbezüglich ist jedoch darauf zu verweisen, dass mit dem α‑Synuklein-RT-QuIC-Verfahren („real time quaking-induced conversion“) bereits ein verbesserter Assay für die zeitnahe Quantifizierung von α‑Synuklein innerhalb von 1 bis 2 Tagen zur Verfügung steht [14]. Beim RT-QuIC-Verfahren handelt es sich um eine Nachweismethode für kleinste Proteinmengen, bei der ein pathogenes Protein eine schnelle Konformationsänderung eines rekombinanten Proteins induziert. Die Bildung dieser Aggregate kann wiederum nachgewiesen werden.

Insbesondere veränderte Konzentrationen der α‑Synuklein-Proteinspezies in Form erniedrigter Gesamt-α-Synuklein-Konzentrationen sowie erhöhter Oligomer- und phosphorylierter α‑Synuklein-Peptide stellen im Rahmen der Synukleinopathien potenziell differenzialdiagnostisch relevante Biomarker im Liquor dar, die bislang jedoch klinisch nicht flächendeckend verfügbar sind [1, 31]. In größeren Untersuchungen konnte gezeigt werden, dass Patienten mit Parkinson-Erkrankungen mittels α‑Synuklein-Spezies mit ausreichender Sensitivität (61–94 %) und Spezifität (25–64 %) von gesunden Kontrollen differenziert werden können [1]. Mit dem neuen RT-QuIC-Assay wird sogar eine diagnostische Sensitivität von 93 % und eine Spezifität von 100 % in der Unterscheidung von Synukleinopathien und Kontrollen erreicht [14].

### Frontotemporale Demenz

Auch im Bereich der 3R- und 4R-Tauopathien und TDP43-Proteinopathien fehlen weiterhin spezifische Biomarker [43]. Insbesondere die Diagnose der behavioralen Variante der frontotemporalen Demenz (bvFTD) und ihre Abgrenzung zu primär psychiatrischen Erkrankungen wie Zwangsstörungen, bipolar affektiven Erkrankungen und Depressionen kann klinisch herausfordernd sein und bedarf, neben einem profunden klinischen Assessment und einer testpsychometrischen Untermauerung, ebenfalls einer umfassenden Biomarkerdiagnostik [8]. Eine wesentliche Aufgabe der Liquordiagnostik bei der bvFTD ist zunächst der Ausschluss einer (atypischen) frontalen Variante der Alzheimer-Erkrankung. Dabei stützt eine isolierte Tau-Protein-Erhöhung die Diagnose einer bvFTD in Abgrenzung zur Alzheimer-Demenz [8]. Ferner scheint die „Aβ42/pTau ratio“ bei der Differenzierung einer FTD von einer Alzheimer-Erkrankung am trennschärfsten zu sein [45].

In der differenzialdiagnostischen Abgrenzung der bvFTD von primär psychiatrischen Erkrankungen könnte das Neurofilament im Liquor ein potenziell bedeutsamer Biomarker sein [8]. Inwieweit der „pTau/Tau ratio“ hier ebenfalls eine diagnostische Bedeutung zukommt, ist Gegenstand aktueller Untersuchungen [8]. Eine neuere Kohortenanalyse zeigte, dass Neurofilament und die „pTau/Tau ratio“ Patienten mit FTD und gesunde Kontrollen gleichermaßen diskriminierten, aber nicht zur Differenzierung der verschiedenen FTD-Entitäten geeignet sind [27].

Neuere Analyseverfahren ermöglichen nun auch den spezifischen Nachweis pathogener Tau-Protein-Formen. Das Tau-Protein kumuliert innerhalb der verschiedenen neurodegenerativen Demenzerkrankungen auf unterschiedliche Weise, was in diesen neueren Analyseverfahren genutzt wird. Während man bei der bvFTD typischerweise 3‑Repeat-Filamente findet (3R), kommen bei der CBD (kortikobasale Degeneration) und PSP typischerweise 4‑Repeat-Filamente (4R) und bei der Alzheimer-Erkrankung ein Gemisch von 3R- und 4R-Filamenten (3R + 4R) vor [34]. Aus diesem Grund ist eine RT-QuIC-Reaktion mit dem 3R-Tau-Filament als Substrat geeignet, die bvFTD von Nicht-bvFTD-Fällen zu unterscheiden [34].

Hinsichtlich weiterer neuer Entwicklungen in der bvFTD-Liquordiagnostik ist außerdem darauf zu verweisen, dass auch das TDP43-Protein ein geeignetes Substrat für eine RT-QuIC-Reaktion ist, womit ein hochsensitives, diagnostisches Nachweisverfahren zur Verfügung steht, was insbesondere in der Frühdiagnostik und Entwicklung verlaufsmodulierender Substanzen hilfreich sein kann [38].

### Creutzfeldt-Jakob-Krankheit (CJD)

**Figure 1:**
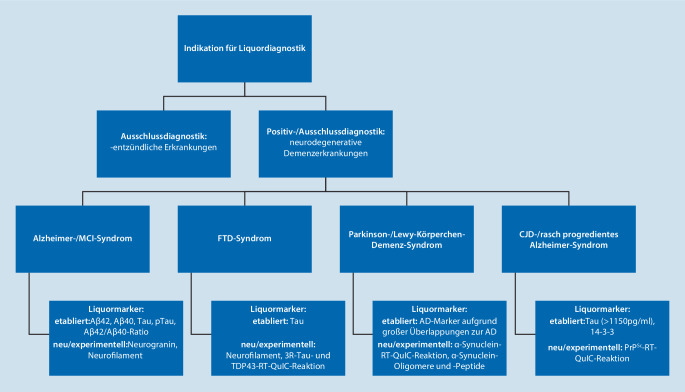

Etablierte Marker der Liquordiagnostik bei Patienten mit CJD sind das Tau- und das 14-3-3-Protein. Das 14-3-3-Protein hat eine diagnostische Sensitivität von 92 % und eine Spezifität von 80 % [28]. Es gilt jedoch zu beachten, dass ein negativer Test die Diagnose nicht ausschließt, was insbesondere für genetische und seltene sporadische Varianten gilt [5, 22]. Hohe Tau-Protein-Konzentrationen von > 1150 pg/ml gelten gegenüber dem 14-3-3-Protein als überlegen [12, 15, 30]. Im Gegensatz zum Tau-Protein werden keine erhöhten pTau-Konzentrationen bei Patienten mit CJD nachgewiesen, weshalb eine erhöhte „Tau/pTau ratio“ eine hohe CJD-Spezifität aufweist [3, 41]. Eine höhere diagnostische Sensitivität und Spezifität wird jedoch mit dem RT-QuIC-Assay erreicht, der das pathogene Prion-Protein (PrP^Sc^) als Substrat verwendet (Sensitivität 92–95 %, Spezifität 98,5–100 %; [13]; Abb. 1).

## Grenzen der Liquordiagnostik

Ein diagnostisches Problem besteht in der begrenzten Vergleichbarkeit von Demenzbiomarkern im Liquor aufgrund der hohen Variabilität der einzelnen Parameter sowie labormethodischer Heterogenitäten in der Analytik und, dadurch bedingt, unzureichend valider Normwerte, insbesondere in präklinischen und prodromalen Demenzstadien [10, 42]. Aus diesem Grund wurde mit dem Erlangen-Score ein diagnostischer Interpretationsalgorithmus entwickelt, der eine norm- bzw. rohwertunabhängige Interpretation und prognostische Einschätzung des Konversionsrisikos erlaubt [10, 23, 42]. Der Erlangen-Score teilt Liquorbefunde bei Patienten mit leichten kognitiven Störungen (MCI) und subjektiven kognitiven Störungen (SCD) in 5 verschiedene Kategorien ein, welche auf nichtdichotome Art und Weise verschiedene Grade und Muster neuropathologischer Veränderungen repräsentieren (graduiert von 0 bis 4, 0 = kein Anhalt für Alzheimer-Erkrankung, 4 = wahrscheinliche Alzheimer-Erkrankung; [10]). Der Erlangen-Score detektiert somit nicht nur die zugrunde liegende Pathologie, sondern ermöglicht auch ein Staging des Erkrankungsprozesses. Studien bestätigen, dass der Erlangen-Score ein geeignetes Instrument darstellt, um das Konversionsrisiko abzuschätzen und auch um eine zugrunde liegende Alzheimer-Erkrankung zu bestätigen [10]. Selbst in Studien mit neuropathologischer Referenz zeigt der Erlangen-Score eine ausreichende diagnostische Sicherheit [42].

Ein weiteres substanzielles Problem in der Liquordiagnostik bei demenziellen Erkrankungen sind Überschneidungen der Biomarkerprofile in der Gruppe primär neurodegenerativer Demenzen (Demenz vom Alzheimer-Typ, Lewy-Körperchen-Demenz, frontotemporale Demenz), was stets die Kontextualisierung von Liquor- zu klinischen Befunden erfordert [46]. Weitere Grenzen der Biomarkerdiagnostik sind, zumindest in bestimmten Regionen, weiterhin fehlende zentrumsübergreifende Methodenstandards und Normwerte für Biomarker der Alzheimer-Erkrankung [33].

## Fazit für die Praxis

- Die Liquordiagnostik ist trotz neuer Methoden im Bereich der metabolischen und funktionellen Bildgebung weiterhin von hoher negativ- und positivdiagnostischer Relevanz im Rahmen der Demenzdiagnostik.
- Die Interpretation von Liquorbefunden bedarf stets der kontextualisierten Bewertung.
- Für die Liquordiagnostik bei Patienten mit MCI existieren keine leitlinienbasierten Empfehlungen.
- Am besten sind liquorchemische Veränderungen im Rahmen der Alzheimer-Erkrankung charakterisiert.
- Unauffällige Liquorbefunde schließen neurodegenerative Demenzursachen mit hoher Wahrscheinlichkeit aus.
- Ein substanzielles Problem sind Überschneidungen der Liquorbiomarkerprofile im Rahmen primär (komorbider) neurodegenerativer Demenzerkrankungen.
- Die Erforschung trennscharfer Liquorbiomarker zur differenzialdiagnostischen Abgrenzung von Tauopathien, TDP43(Transactive response DNA binding protein 43 kDa)-Proteinopathien und Synukleinopathien ist Gegenstand aktueller Untersuchungen.
- Insbesondere die Interpretation von Mischbefunden bedarf profunder präanalytischer und klinisch-diagnostischer Kenntnisse.
